# Lipid‐coated bismuth nanoflower as the thermos‐radio sensiti for therapy of lung metastatic breast cancer: Preparation, optimisation, and characterisation

**DOI:** 10.1049/nbt2.12097

**Published:** 2022-08-29

**Authors:** Shushu Xue, Junrong Jiao, Si Miao, Lijun Wang, Yang Liu, Qingjie Zhang, Qiyue Wang, Yu Xi, Yuanyuan Zhang

**Affiliations:** ^1^ Department of Radiotherapy Jiangsu Cancer Hospital Jiangsu Institute of Cancer Research The Affiliated Cancer Hospital of Nanjing Medical University Nanjing China; ^2^ Department of Pharmaceutics State Key Laboratory of Nature Medicines China Pharmaceutical University Nanjing China; ^3^ School of Pharmaceutical Science Nanjing Tech University Nanjing China; ^4^ Department of Pharmacy Jiangsu Cancer Hospital Jiangsu Institute of Cancer Research The Affiliated Cancer Hospital of Nanjing Medical University Nanjing China

**Keywords:** bismuth nanoflower, dry powder inhalation, lung metastatic breast cancer, optimisation, radiosensitiser

## Abstract

Lung metastatic breast cancer (LMBC) leads to a large number of deaths in women with breast cancer, and radiotherapy has been considered the common assay for tumour therapy except for surgery. However, radiotherapy still faces problems of low efficiency due to resistance and easily induced side effects. Here, the authors designed lipid‐decorated bismuth‐based nanoflowers (DP‐BNFs) as both a radiosensitiser and a photothermal therapy agent for LMBC treatment. The BNFs were prepared by oxidation of bismuth nitrate and subsequent reduction using sodium borohydride. The preparation parameters and formulation of DP‐BNFs were optimised via a single‐factor experiment, with the factors including reaction temperature, a molar ratio of reducing agents, and the types and amount of decorated lipid materials. The result indicated that the BNFs prepared at 170°C with the Bi/NaBH_4_ ratio of 1:0.7 exhibited the best yield and particle size around 160 nm. After being spray dried with lactose to prepare dry powder inhalation (DP‐BNF@Lat‐MPs), their effects on improving therapeutic efficiency of the radiotherapy and photothermal therapy combination were measured using the western blot assay to determine the tumour apoptosis. In a word, DP‐BNF@Lat‐MPs could be a novel inhalable integrated microsphere that provides a new possibility for thermoradiotherapy of LMBC.

## 1 INTRODUCTION

Although early diagnosis and treatment can effectively improve the survival rate, breast cancer is still the most common cancer in women with the second highest death rate [1, 2]. Among them, metastatic breast cancer leads to almost 90% of deaths [3]. The metastasis of breast cancer exhibits an organ tendency that mainly metastasises to bone tissue, brain tissue, liver, and lung. Lung metastatic breast cancer (LMBC) accounts for 20% of total organ metastasis with a mortality rate of over 60% [4, 5]; therefore, effective treatment strategies need to be developed to improve the poor therapeutic efficacy. Radiotherapy has been considered the most commonly used tumour therapeutic method. The ionising radiation generated by high‐energy rays such as X‐rays and γ‐rays could act on tumour lesions followed by inducing tumour cell damage and apoptosis [6]. However, radiotherapy still faces some disadvantages that affect the treatment efficiency such as easily damaging the healthy tissues surrounding the tumour lesion and a hypoxic environment leading to radiotherapy resistance [7]. How to accurately and effectively introduce an appropriate radiation dose into the tumour lesion area to eliminate the tumour tissues without obvious side effects is still a major problem for radiotherapy development. Recently, several types of radiosensitisers have been developed and applicated in clinical trials, which brings hopes for a solution to this issue [7, 8, 9, 10, 11].

The development of nanotechnology has promoted the discovery and development of effective radiosensitisers. The heavy metal nanomaterials with high atomic numbers show broad prospects as radiosensitisers because of their ability to absorb and transform radiate energy, which has become a research hotspot of novel radiosensitisers development [12, 13]. Of the variety of metal materials, bismuth (Bi) has been considered the potential material for radiosensitiser development [14, 15, 16]. The x‐ray absorption coefficient has been confirmed to be proportional to the atomic number of the material. Bi with a high atomic number can effectively generate secondary electrons under the excitation of x‐ray followed by generating free radicals such as reactive oxygen species (ROS), which improves the sensitivity of radiotherapy [17, 18]. Moreover, Bi also exhibits excellent photothermal conversion ability, suggesting the potential to be a photothermal therapy (PTT) sensitiser.

Systemic delivery still is the most common route for delivering active agents to the target area. Even with the advantages of rapid delivery and no need for absorption, intravenous injection still faces problems such as low patient compliance, systemic toxicity, and frequent administration [19]. Pulmonary administration is an alternative drug delivery route that exhibits advantages in local drug delivery. The pulmonary delivery of gold nanoparticles, cisplatin nanoparticles, and carboplatin nanoparticles has been investigated, which confirms that the lung accumulation of drugs by pulmonary delivery is always 3.6–14.6 times higher than intravenous injection, which also reduces the unnecessary biodistribution and systemic side effects [20, 21]. To develop suitable pulmonary delivery formulations for Bi‐based materials, administration is an urgent need to expand the application of Bi‐based materials in cancer radiotherapy and PTT.

A dry powder inhaler could deliver the solid form of active agents into the lung with a specific device. Due to the high stability and targetability, the development of dry powder inhalation (DPI) has been considered a research hotspot for pulmonary local drug delivery. A successful DPI requires suitable aerodynamic diameters ranging from 1 to 5 μm to achieve effective lung deposit [22]. Moreover, the particle shapes, hygroscopicity, and surface morphology also strongly affect the effective deposition of active agents by affecting the flowability of the powder. Therefore, the lactose‐based microparticles were developed as the inhalation carriers to load drugs or nanoparticles for effective pulmonary delivery [23, 24].

In this research, lipid‐decorated bismuth nanoflowers (DP‐BNFs) were developed for the combination therapy of radiotherapy and PTT on LMBC. The formulation and preparation of BNFs and DP‐BNFs were optimised with the particle size as the key indicator. The inhalable DP‐BNFs‐loaded lactose microparticles (DP‐BNF@Lat‐MPs) were prepared by spray drying and characterised by the size, morphology, and stability with the median mass aerodynamic diameter (MMAD) and fine particle fraction (FPF) as the key parameters. The therapeutic efficiency of radiotherapy and PTT with DP‐BNF@Lat‐MPs as a dual sensitiser on LMBC were investigated using the western blot assay to measure the key proteins of apoptosis. This study proposed a promising formulation and paved the way to develop Bi‐based metal materials for pulmonary drug delivery.

## 2 MATERIALS AND METHODS

### 2.1 Materials and cells

Bi(NO_3_)_3_·5H_2_O and NaBH_4_ were purchased from Aladdin Biochemical Technology Co., Ltd. 1,2‐distearoyl‐sn‐glycero‐3‐phosphorylethanolamine (DSPE) and phosphatidylcholine (PC) were purchased from A.V.T Pharmaceutical Co., Ltd. Other chemicals were purchased from Xilong Scientific Co., Ltd.

4T1 breast cancer cells were purchased from KeyGEN Bio TECH Co., Ltd. and cultured in RPMI 1640 complete media supplemented with 10% foetal bovine serum, 2 mM of L‐Glutamine, and 1% of the penicillin‐streptomycin solution. Cells were maintained at 37°C in a humidified atmosphere with 5% CO_2_. RIPA Lysis Buffer and BCA protein assay kit were purchased from Beyotime Biotechnology Co., Ltd. 3‐(4,5‐dimethylthiazol‐2‐yl)‐2,5‐diphenyl tetrazolium bromide (MTT) was purchased from Jiangsu KeyGEN BioTECH Co., Ltd. Anti‐Cleaved Caspase 3 antibody [E83‐77], anti‐HSP‐70 antibody [5A5], and anti‐p‐p65 antibody [EP2294Y] were purchased from Abcam.

### 2.2 Preparation of BNFs

To prepare the BNFs, bismuth oxide (Bi_2_O_3_) was firstly synthesised via hydrothermal reaction and then partly reduced using sodium borohydride. Briefly, Bi(NO_3_)_3_·5H_2_O (0.75 mmol) were dissolved in 10 ml of HNO_3_ solution (1 M) under sonication, followed by mixing with 2.7 mmol of NaOH and 50 ml of PVP‐K30 ethylene glycol solution (*w*/*v*, 2.5%). The mixture was then transferred into a polytetrafluoroethylene‐coated airtight container (Kunshan Ultrasonic Instrument Co. Ltd.) and was made to react for 3 h under 170°C. The synthesised Bi_2_O_3_ NPs were collected via centrifugation (HC‐3018, Anhui USTC Zonkia Scientific Instrument Co., Ltd.) at 10,000 rpm for 30 min, followed by purification by washing it thrice using DI water. Then, 2 ml of NaBH_4_ solution (5 mg/ml) were added dropwise into the Bi_2_O_3_ NPs suspension and the reaction continued for another 1 h at room temperature under stirring. Finally, the BNFs were collected by centrifugation of the mixture at 10,000 rpm for 30 min followed by purification by washing it thrice using DI water.

### 2.3 Preparation of DP‐BNFs

1,2‐distearoyl‐sn‐glycero‐3‐phosphorylethanolamine (DSPE) and PC were used as the lipid materials for decoration of the BNFs using the film‐hydration method. Briefly, DSPE and PC were dissolved in the chloroform‐ethanol (2:1) mixture and the thin lipid film was prepared by evaporation of the organic solvents under the vacuum. The warmed phosphate buffered saline (PBS) containing BNFs was added to prepare the DP‐BNFs via vortexing and stirring for 30 min. The DP‐BNF particle size were further minimised using the Mini‐Extruder Extrusion Technique [15] via extruding DP‐BNFs through 200 nm polycarbonate porous membranes five times.

### 2.4 Optimisation of BNFs and DP‐BNFs preparation

#### 2.4.1 Effects of reaction temperature

To investigate the effects of reaction temperature on the preparation of BNFs, the mixture was made to react for 3 h under 150, 170, and 190°C, respectively, followed by the purification process and reducing reaction described in Section 2.2. The particle size and zeta potential were then measured.

#### 2.4.2 Effects of stirring rate

To investigate the effects of stirring rate on the preparation of BNFs, the reducing reaction was stirred under 650, 780, and 950 rpm, respectively, followed by the purification process described in Section 2.2. The particle size and zeta potential were then measured.

#### 2.4.3 Effects of reducing agent ratio

To investigate the effects of reducing agent ratio on the preparation of BNFs, we designed the reaction with a series of molar ratios of Bi(NO_3_)_3_·5H_2_O and NaBH_4_ (0.55:1, 0.6:1, 0.65:1, and 0.7:1) as reacting agents, respectively. The followed synthesis process was described in Section 2.2. The particle size and zeta potential were then measured.

#### 2.4.4 Optimise the lipid prescription and ratio of lipid materials and BNFs

The lipid composition used for decoration of BNFs was further investigated by adjusting the ratio of DSPE and PC. The DSPE and PC with a molar ratio of 1:3, 1:6, 1:9, and 1:12 were dissolved in the organic solvent, respectively, followed by evaporation to prepare the lipid film. The DP‐BNFs were then formulated following the description in Section 2.3.

Moreover, the mass ratio of lipid materials and BNFs was also investigated. The PBS solution with different masses of BNFs dispersed was added into the lipid film (Lipid:BNF of 3:1, 6:1, or 9:1) followed by preparing the DP‐BNFs, respectively. The DP‐BNFs were then formulated following the description in Section 2.3. The particle size and zeta potential were then measured.

### 2.5 Characterisation of DP‐BNFs

The particle size and zeta‐potential of BNFs and DP‐BNFs were measured by dynamic light scattering using a Zerasizer Nano‐ZS90 (Malvern Panalytical Ltd.). To determine the morphology of BNFs and DP‐BNFs by transmission electron microscope (TEM), particles were diluted at a suitable concentration and TEM imaging was carried out using an HT7700 TEM (Hitachi Ltd.).

### 2.6 Preparation and characterisation of DP‐BNF@Lat‐MPs

#### 2.6.1 Preparation of DP‐BNF@Lat‐MPs

For the further preparation of DP‐BNF@Lat‐MPs, 3 g of DP‐BNFs, 3 g of lactose, and amino acids (1 g of arginine and 1 g of leucine) were dissolved in DI water followed by spray drying (ADL311SA, Yamato Scientific America) under the optimised conditions described below: the inlet temperature of 150°C, the feeding rate of 0.17 l/h, and the content concentration of 1%.

#### 2.6.2 Morphology

The morphology of DP‐BNF@Lat‐MPs was measured using a Hitachi SU8010 scanning electron microscope (SEM, Hitachi Ltd.). Particles were sprinkled onto a silicon stub followed by sputter coating with gold and observed under SEM at 15 kV.

#### 2.6.3 Content uniformity

The content of Bi in the DP‐BNF@Lat‐MPs powder was measured by inductively coupled plasma mass spectrometry (ICP‐MS). The DP‐BNF@Lat‐MPs powder was filled into hydroxypropyl methylcellulose capsules with a dose of 10 mg. The content uniformity was measured by dissolving into DI water followed by measuring via ICP‐MS.

#### 2.6.4 Dumping rate

The dumping rate of DP‐BNF@Lat‐MPs‐loaded capsules was measured using an NGI (Copley Scientific Ltd.). The NGI was assembled with an induction port and an artificial throat followed by adjusting the airflow rate by a vacuum pump (HCP5, Copley Scientific Ltd.) and airflow meter (TPK 2000, Copley Scientific Ltd.). The capsules loaded with DP‐BNF@Lat‐MPs powders (10 mg) were fixed in a dry powder inhaler (DPI, KRT‐D01, Charamedical) followed by release into the NGI at an airflow rate of 60 l/min with a conducting time of 1.5 s. The suction process for each capsule was repeated four times and the dump rate was calculated by measuring the weight differences before and after the suction.

#### 2.6.5 Stability

The stability of DP‐BNF@Lat‐MPs‐loaded capsules was investigated under the thermal stress condition (40°C and 75% relative humidity [RH]), humidity stress condition (25°C and 90% RH), and photo light condition (25°C, 75% RH, and 4500 Lux). DP‐BNF@Lat‐MPs‐loaded capsules were placed under different conditions for 10 days and their appearance and aerodynamic performance were evaluated using NGI.

### 2.7 Haemolysis test

The haemolysis test of DP‐BNF@Lat‐MPs was investigated to examine the toxicity of Bi‐based materials. Fresh blood from rats was collected (12 ml) followed by mixing with an addition of 0.4 ml of 20 g/l potassium oxalate reagent and was then diluted to obtain a 2% red blood cell suspension using saline solution. DP‐BNF@Lat‐MPs were dissolved into a 0.9% normal saline solution and further diluted to 4 mg/ml stocking solution. A series concentration of DP‐BNF@Lat‐MPs solution was incubated with a 2.5 ml of 2% red blood cell suspension for the haemolysis tests. The deionised water and a 0.9% saline solution were used as positive and negative control, respectively. All solution was mixed and incubated at 37°C for 2 h, respectively. The supernatant was collected after being centrifuged at 1,500 rpm for 5 min. The absorbance values were measured by a multimeter (Multiskan MK3, Thermo Fisher Scientific) at a wavelength of 570 nm and the haemolysis percentages were calculated according to the formula list below:

$$\text{Hemolysis}\;\text{rate}\;\left(\%\right)=\frac{A_{t}-A_{nc}}{A_{pc}-A_{nc}}\times 100\;\%$$

where *A*_*t*_, *A*_*nc*_, and *A*_*pc*_ represent the absorbance of testing groups, negative control, and positive control, respectively.

### 2.8 Western blot assay

LMBC‐bearing mice model was built following our previous research and the mice were separated into six groups: (I) Control group; (II) PBS Near‐intrared (NIR) group; (III) PBS IR group; (IV) DP‐BNF@Lat‐MPs NIR group; (V) DP‐BNF@Lat‐MPs IR group; and (VI) DP‐BNF@Lat‐MPs NIR + IR group. For the groups (I–III), mice were administered PBS by intratracheal insufflation while mice in groups (IV–VI) were administered DP‐BNF@Lat‐MPs (50 mg/kg) every 3 days for 5 times. The mice then received NIR (3 W/cm^2^, 5 min) and IR (10 Gy), respectively, or both irradiation at 30 min post administration. After the therapeutic process, mice in each group were euthanised and the tumour nodules in the lung were separated for total protein extraction using ReadyPrep™ Protein Extraction Kit. After separating sodium dodecyl sulphate‐polyacrylamide gel electrophoresis (SDS‐PAGE), the proteins were then transferred to nitrocellulose membranes and fixed followed by incubation with primary antibodies at 4°C overnight and secondary antibodies at room temperature for 1 h. The membranes were illuminated using enhanced chemiluminescence kits and imaged using a gel imaging system.

### 2.9 Statistical analysis

In this study, the experimental results are expressed as mean ± standard deviations (SD). Student's *t*‐test (comparing only two individual groups) and One‐Way ANOVA (comparing more than two groups) were applied to determine the statistical significance with a minimum confidence level of 0.05.

## 3 RESULTS AND DISCUSSION

### 3.1 Optimisation of BNFs and DP‐BNFs preparation

#### 3.1.1 Effects of reaction temperature

**FIGURE 1:**
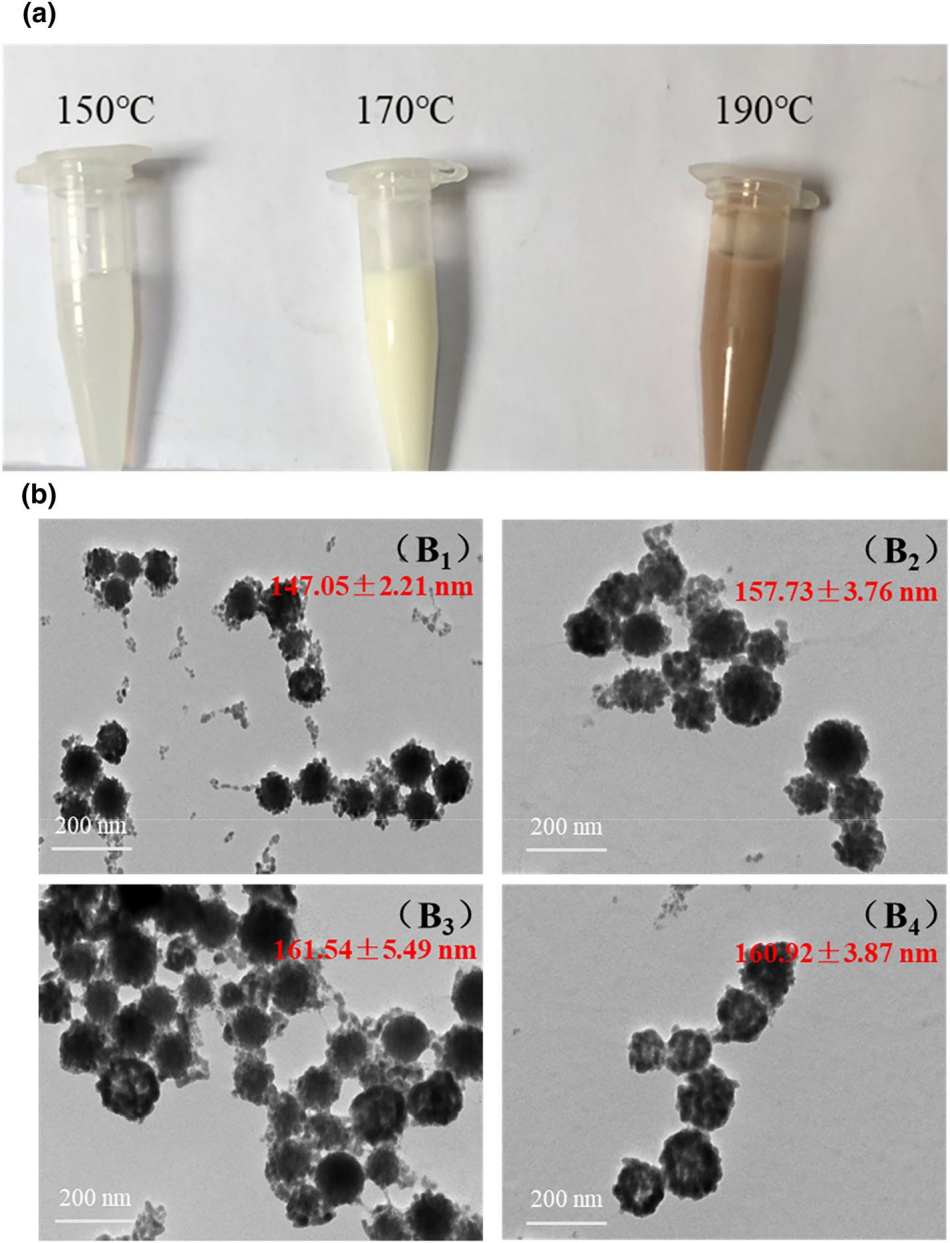
(a) The appearance of Bi_2_O_3_ nanoparticles with different reaction temperatures. Transmission electron microscope images of BNFs prepared with the NaBH_4_/Bi_2_O_3_ ratio of (b_1_) 0.55; (b_2_): 0.60; (b_3_): 0.65; (b_4_): 0.70

To investigate the effect of reaction temperature on BNFs, Bi_2_O_3_ was prepared at 150, 170, and 190°C, respectively. The particle size and zeta potential data are shown in Table 1. The reaction temperature at 150°C was not high enough to boost the reaction with no Bi_2_O_3_ formation and the reacting solution kept clear. At the reaction temperature of 170°C, a milky white bismuth oxide solution was obtained for further reduction. While with the reaction temperature at 190°C, the bismuth was over‐oxidised, which exhibited a brownish yellow colour (Figure 1a). After adding sodium borohydride to reduce the above bismuth oxide solution, the particle size and zeta potential were measured. The bismuth oxide solution prepared at 150°C hardly collected the black BNFs, while the bismuth oxide solution prepared at 190°C easily aggregated with a large particle size of over 500 nm. Only when the reaction temperature was at 170°C, the bismuth oxide solution could gradually transform into a grey‐black solution after reduction with good dispersion ability. The particle size and zeta potential of the obtained BNFs were 160.03 nm and −17.25 mV (Table 1), respectively, indicating that the preparation of bismuth nanoflowers was affected sensitively by the temperature and the optimised reaction temperature was chosen at 170°C.

**TABLE 1:** The particle size and zeta potential of BNFs were prepared at different temperatures

Temperature (°C)	Diameter (nm)	Polydispersity Index (PDI)	Zeta potential (mV)
150	/	/	/
170	160.03 ± 3.23	0.156 ± 0.02	−17.26 ± 0.45
190	560.53 ± 24.32	0.47 ± 0.02	/

Abbreviation: PDI, Polydispersity Index.

#### 3.1.2 Effects of stirring rate

To investigate the effect of stirring speed, BNFs were prepared at stirring speeds of 650, 780, and 950 rpm, respectively. The particle size of BNFs is about 296.93 nm with a stirring speed of 650 rpm, which is too large and can easily sediment. With a stirring speed of 950 rpm, the reaction product directly aggregated at the bottom of flask and particle size could not be measured. Only with the stirring speed of 780 rpm, the BNFs were prepared and it exhibited a suitable particle size of 159.03 ± 2.23 nm and the potential of −17.10 mV (Table 2). The results indicated that the stirring rate also could affect the BNF generation and the stirring speed of 780 rpm was chosen as the optimised conditions.

**TABLE 2:** The particle size and zeta potential of BNFs were prepared with different stirring speeds

Stirring speed (rpm)	Diameter (nm)	PDI	Zeta potential (mV)
650	296.93 ± 3.51	0.27 ± 0.12	/
780	159.03 ± 2.23	0.14 ± 0.05	−17.10 ± 0.96
950	/	/	/

Abbreviation: BNF, bismuth‐based nanoflower.

#### 3.1.3 Effects of reducing agent ratio

To study the effect of the reducing agent ratio on the morphology, we prepared BNFs with different NaBH_4_/Bi_2_O_3_ ratios of 0.55, 0.60, 0.65, and 0.7. The morphology of BNFs was observed by TEM. As shown in Figure 1b, with the increment of the reducing agent, the outer edge of BNFs exhibited a slight transition from a smooth to rough edge. As the roughness of nanoparticles could improve the photothermal conversion efficiency, we selected the NaBH_4_/Bi_2_O_3_ ratios of 0.7 for the preparation of BNFs.

#### 3.1.4 Effects of lipid prescription

**FIGURE 2:**
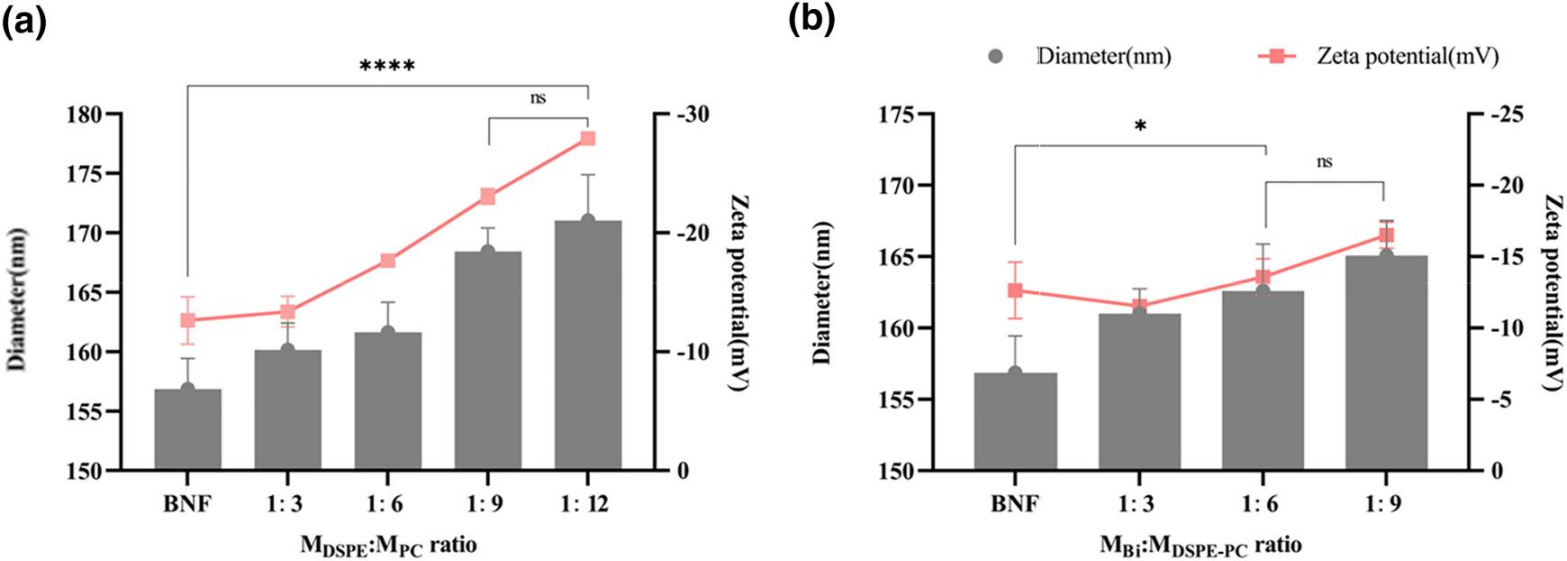
The diameter and zeta potential of BNFs with (a) different mass ratios of Bi and lipid materials and (b) different mass ratios of DSPE and PC in the total lipid (****p < 0.0001, *p < 0.05, n.s. *p* > 0.5). DSPE, 1,2‐distearoyl‐sn‐glycero‐3‐phosphorylethanolamine; PC, phosphatidylcholine

The lipid out layer modification could improve the stability of BNFs by improving the hydrophilicity of particle surface, which could reduce the protein crown formation and clearance by macrophages [25]. Therefore, the lipid ingredient would be investigated to optimise the thickness of the surface layer. As shown in Figure 2a, the particle size was increased consistently with the content increment of the PC in the lipid materials. This increment was due to the lipid‐coated outside of the BNFs. However, there was no significant difference in particle size between the DSPE/PC ratio of 1:9 and 1:12, indicating that the BNFs have been integral coated, suggesting the ratio of DSPE/PC of 1:9 should be the optimised lipid layer composition.

Furthermore, we investigate the lipid amount that should be used in the BNF coating. With the increment of lipid, the particle size of DP‐BNFs significantly increased from 158 to 164 nm when the mass ratio of Bi/lipid was 1:6 (Figure 2b). The zeta potential also decreased as the lipid coating reached −14.66 mV. There was no significant difference in particle size between the ratio of 1:6 and 1:9, suggesting that the ratio of 1:6 should be the optimised amount of lipid in DP‐BNFs preparation.

### 3.2 Particle size and morphology of DP‐BNF@Lat‐MPs

To achieve effective lung deposition, the DP‐BNFs should be loaded to inhalable carriers to achieve the suitable MMAD. Here, we use the spray‐drying method to prepare the DP‐BNFs co‐loaded lactose microparticles. The feed concentration of lactose was investigated. With the increase in feeding concentration from 1% to 5%, the yield of microparticles slightly decreased from 36% to 32% (Table 3). The most important changes were observed in the particle size. The D_90_ of DP‐BNF@Lat‐MPs was increased to 19.65 ± 0.16 μm and 51.25 ± 0.09 μm when the lactose feeding concentration was 3% and 5%, respectively, which is too large and hardly delivers DP‐BNFs into pulmonary by inhalation. This data suggesting that the 1% of feeding concentration was suitable for preparation of DP‐BNF@Lat‐MPs.

**TABLE 3:** The characterisation of DP‐BNF@Lat‐MPs.

Lactose concentration (%)	Yield (%)	Appearance	D_10_ (μm)	D_50_ (μm)	D_90_ (μm)	Span
1	36.37	White powder	1.36 ± 0.08	2.89 ± 0.02	5.31 ± 0.04	1.45 ± 0.05
3	35.86	White powder	2.39 ± 0.07	6.51 ± 0.03	19.65 ± 0.16	2.65 ± 0.01
5	32.23	White powder	2.08 ± 0.12	5.93 ± 0.20	51.25 ± 0.09	8.29 ± 0.10

**FIGURE 3:**
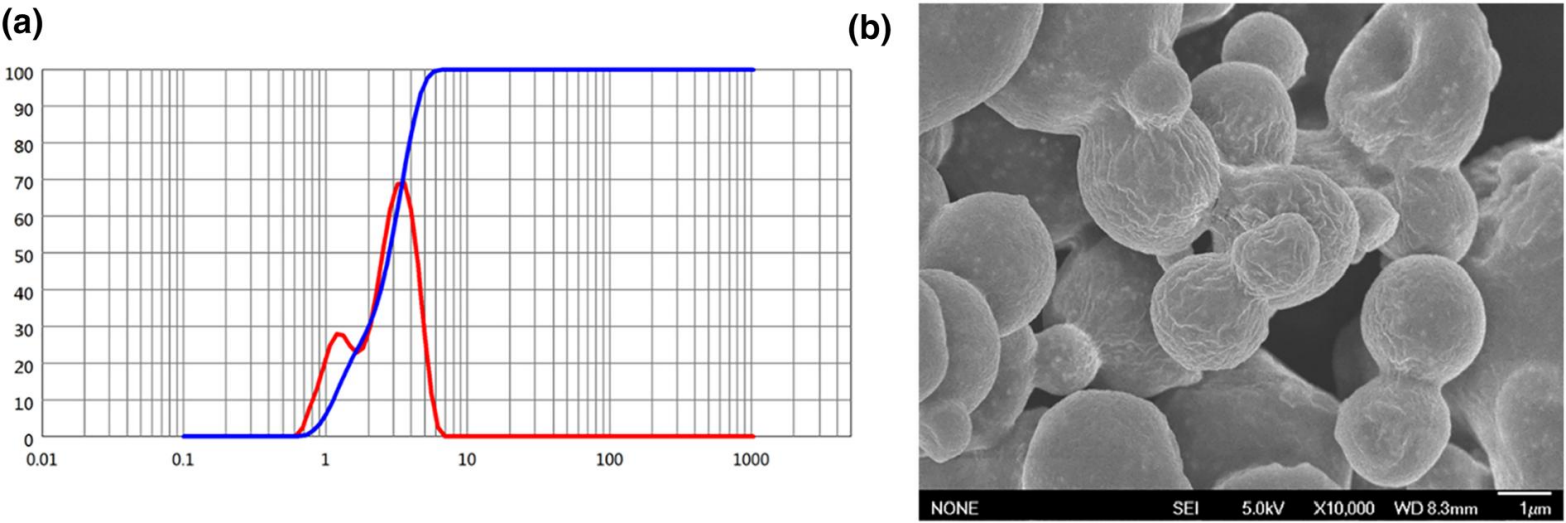
The (a) diameter and (b) scanning electron microscopy image of DP‐BNF@Lat‐MPs

Using the optimised parameters, we formulated the DP‐BNF@Lat‐MPs for further investigation. The particle size of microparticles was shown in Figure 3a. The particle D_90_ of DP‐BNF@Lat‐MPs was around 5 μm, indicating that 90% of particles were suitable for effective lung deposition. Moreover, the morphology of DP‐BNF@Lat‐MPs was observed by SEM. The surface of the microparticle was rough due to the additional amino acid in the formulation. The amino acid with less water solubility was firstly precipitated during the spray‐drying process and formed the amino acid protective shell. This hydrophobic shell could also reduce the humidity diffusion and improve the moisture resistance of DPI [26, 27]. Moreover, previous research proposed that particles with a rough surface are more conducive to lung deposition because the adhesion between particles was negatively correlated with the roughness of the particles, indicating that the particles with a higher rough surface could exhibit better dispersibility during the aerosol inhalation.

### 3.3 Content uniformity

Content uniformity is a pharmaceutical analysis parameter for the quality control of capsules to assay the individual content of the active ingredient in each capsule. The preparation complies if no more than one individual content is beyond the limit of 85%–115% of the average content. The content of DP‐BNF@Lat‐MPs in 10 random capsules was measured by ICP‐MS to confirm content uniformity. As shown in Table 4, the content of Bi was above 96% of theoretical content with the SD lower than 1.5%, indicating that the prepared DP‐BNF@Lat‐MPs show excellent content uniformity.

**TABLE 4:** Content uniformity evaluation and dumping ratio of DP‐BNF@Lat‐MPs

Content (%)	Average content (mean ± SD) (%)	Dumping ratio (%)	Average (mean ± SD) (%)
99.33	99.23 ± 1.14	94.50	95.18 ± 2.69
96.98		94.95	
99.30		91.84	
100.13		98.82	
100.21		98.99	
100.44		95.88	
98.61		93.52	
100.67		96.74	
98.84		90.72	
97.78		95.83	

### 3.4 Dumping rate

The dumping rate of DP‐BNF@Lat‐MPs‐loaded capsules was measured using a new generation of the pharmaceutical impactor (NGI). As shown in Table 4, the average dumping rate was 95.18% ± 2.69%, which complies with the relevant regulations of the Chinese Pharmacopoeia 2020 Edition on the emptying rate. The data exhibit that the DP‐BNF@Lat‐MPs exhibited good emptying performance, which could successfully deliver active agents into the lung by inhalation.

### 3.5 Stability

The stability data of DP‐BNF@Lat‐MPs were exhibited in Table 5. The aerosol performance of microparticle powder deteriorated under the high‐temperature condition, with the FPF and dumping rate significantly decreased. The same situation has been observed under the high‐humidity condition with the powder agglomeration and loss of dispersion ability. The DP‐BNF@Lat‐MPs stored in the strong light condition exhibited no difference in dumping rate and MMAD on the fifth day and only exhibited a slight decrease of FPF. While the same deterioration happened on day 10 with the significant decrement of FPF and dumping rate and increased MMAD. The stability data confirm that the RH had the greatest effect on the properties of DP‐BNF@Lat‐MPs and the temperature also could infect the stability of dry powder. Therefore, DP‐BNF@Lat‐MPs should be stored in a dry and cool place at room temperature and avoid strong light.

**TABLE 5:** The stability of DP‐BNF@Lat‐MPs

	Time (day)	FPF (%)	Dumping ratio (%)	MMAD (μm)	Appearance
High temperature	0	48.41 ± 2.81	95.18 ± 2.69	5.15 ± 0.21	Good disperse
	5	37.69 ± 2.45	70.45 ± 6.50	5.907 ± 1.23	Good disperse
	10	/	/	/	Agglomerate
High humidity	0	48.41 ± 2.81	95.18 ± 2.69	5.15 ± 0.21	Good disperse
	5	/	/	/	Agglomerate
	10	/	/	/	Agglomerate
Strong light radiation	0	48.41 ± 2.81	95.18 ± 2.69	5.15 ± 0.21	Good disperse
	5	41.31 ± 2.45	92.41 ± 1.65	5.65 ± 2.21	Good disperse
	10	30.91 ± 1.62	90.85 ± 2.40	6.38 ± 2.14	Good disperse

Abbreviations: FPF, fine particle fraction; MMAD, median mass aerodynamic diameter.

### 3.6 Haemolysis test

**FIGURE 4:**
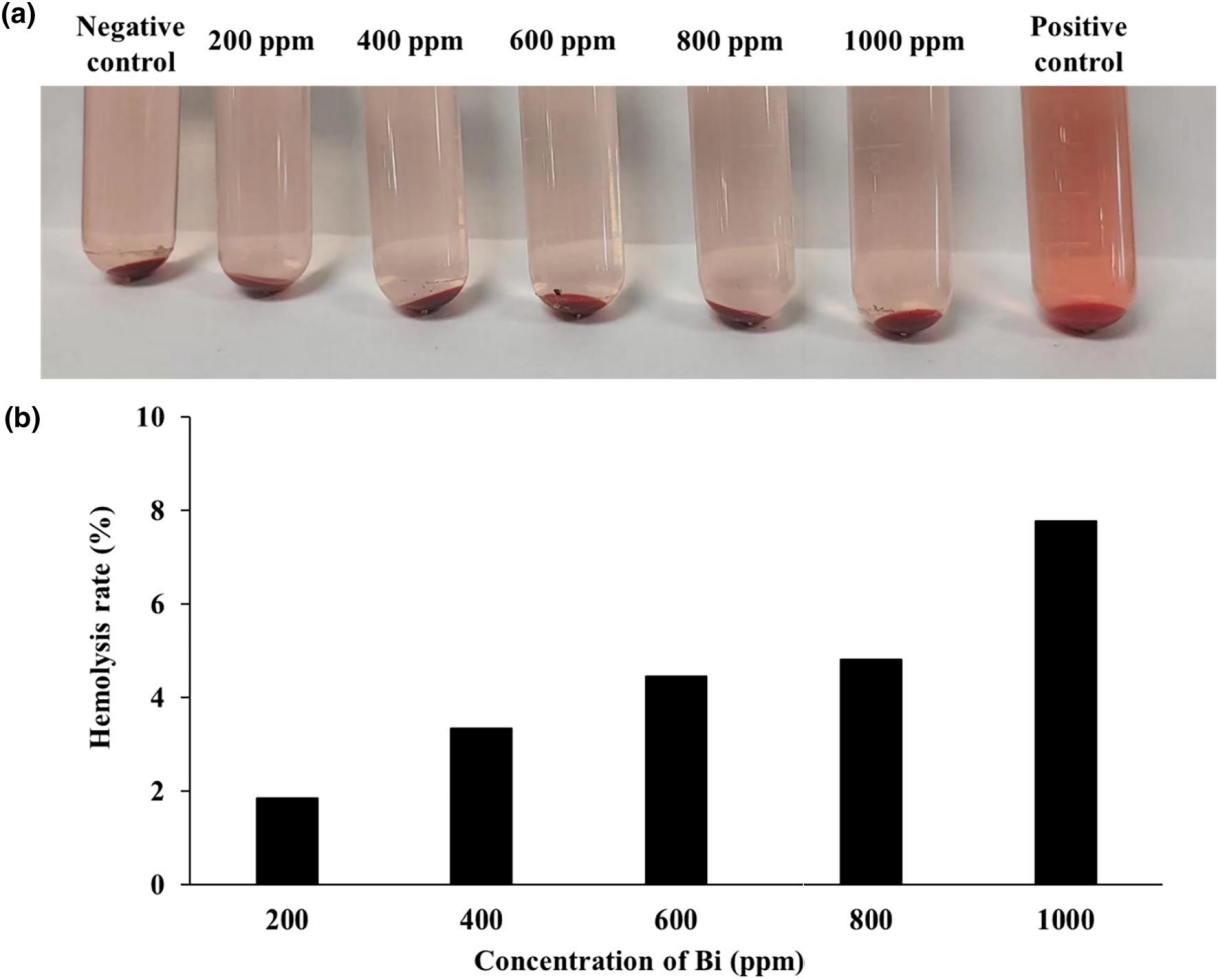
The picture of (a) haemolysis tests and the (b) haemolysis rate of DP‐BNF@Lat‐MPs

The haemolysis test was used to verify the biosafety of DP‐BNF@Lat‐MPs. The red blood cells were incubated with different concentrations of DP‐BNF@Lat‐MPs at 37°C for 2 h. As shown in Figure 4, the DP‐BNF@Lat‐MPs with Bi concentrations between 200 and 1000 ppm had not induced lysis of red blood cells with a clear and colourless supernatant compared to the negative control, whereas the positive control exhibited the obvious red colour of supernatant indicating the haemolysis of red blood cells. Moreover, we measured the absorbance of supernatant of each group at a wavelength of 570 nm to calculate the haemolysis rate. The haemolysis rate exhibited a concentration‐dependent increase and the group with 1000 ppm of Bi exhibited the highest haemolysis rate (7.7%), which still meets the requirement that the haemolysis rate should be less than 10% for material safety, indicating that DP‐BNF@Lat‐MPs has good biological safety.

### 3.7 Western blot assay

**FIGURE 5:**
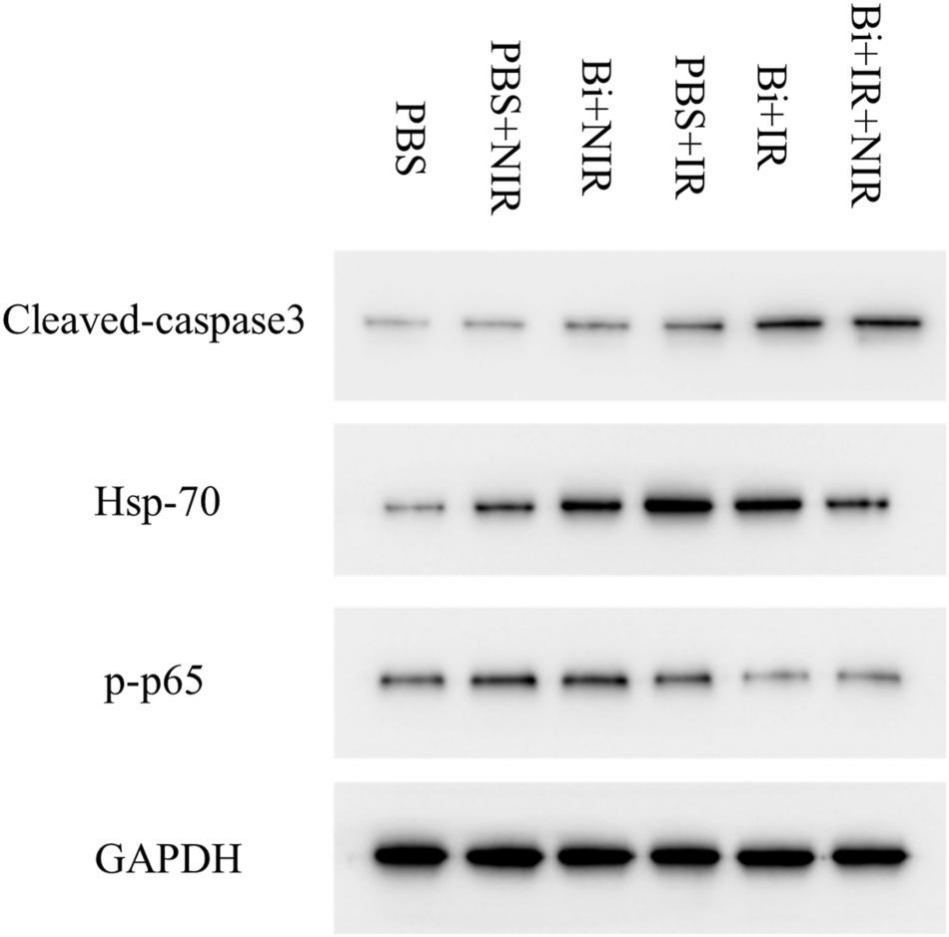
Western Blot analysis of the expression of p‐p65, HSP70, and Cleaved Caspase‐3 in tumour nodules

The expression of Cleaved Caspase‐3, HSP70, and p‐p65 proteins in each group was determined and shown in Figure 5. Cysteine proteases (Caspases) are a family of proteases and are key mediators of programmed cell death or apoptosis. One specific effector of caspase is caspase‐3, a protein that is cleaved and thus activated upon the initiation of apoptosis [27]. Cleaved caspase‐3 propagates an apoptotic signal through enzymatic activity on downstream targets, including poly ADP ribose polymerase (PARP) and other substrates, which are therefore detected as strong indicators of cell death induction. As shown in Figure 5, the cleaved caspase three expression was significantly increased in all groups compared to the control group (PBS alone), suggesting that the tumour treated with NIR and x‐ray irradiation could strongly induce tumour cell death. However, the cleaved caspase three expression in Bi + IR and Bi + IR + NIR groups looks lower than in other therapeutic groups, which may be due to the strongest cytotoxicity effects of IR and NIR + IR groups with DP‐BNF@Lat‐MPs as a sensitiser. The cleaved caspase 3 was only activated in the early apoptosis stages. Their lower expression suggests that the tumour in these groups has almost all been moved into the late apoptotic stage.

A similar expression trend has been found in HSP‐70 expression. The heat shock protein HSP70 is a signal that awakens the body's anti‐tumour immunity [28]. Under stress conditions such as radiotherapy and ROS generated by hyperthermia, the tumour cells can induce the generation of HSP70 and release it into the tumour microenvironment, thereby promoting tumour cell recognition and apoptosis. As shown in Figure 5, the expression of HSP70 in the remaining treatment groups significantly increased compared with the control group. Their lower expression in Bi + IR and Bi + IR + NIR groups indicates that radiotherapy could reverse the thermal resistance of tumour cells caused by PTT, which could synergistically improve tumour apoptosis in the combination therapy.

The p65 protein is a subunit of the NF‐κB protein. With the tumour cells in the state of oxidative stress (ROS), radiation therapy, or chemotherapy, the NF‐κB signalling pathway was activated followed by reducing the radiation sensitivity of tumour cells and inhibiting the apoptosis of tumour cells [29]. As shown in Figure 5, compared with the control group, the expression of p‐p65 in PBS + NIR and Bi + NIR groups was increased indicating that ROS generated by PTT can activate the NF‐κB signalling pathway. While for radiotherapy, the expression of p‐p65 in the PBS + IR group was also increased compared to the control groups, confirming that the x‐ray irradiation also could induce activation of the NF‐κB signalling pathway. In the Bi + IR and Bi + IR + NIR groups, the expression of NF‐κB was decreased compared to other therapeutic groups and only slightly higher than that in the control group, indicating that the DP‐BNF@Lat‐MPs could improve the radiosensitivity of tumour cells and improve the therapeutic effect of radiotherapy.

## 4 CONCLUSION

In this study, BNFs were modified with lipid materials and co‐sprayed with lactose, to prepare inhaled macroparticles for the LMBC diagnosis and thermoradiotherapy. The preparation process and the formulation of DP‐BNFs were optimised and the therapeutic efficiency was measured using the western blot assay. The DP‐BNF@Lat‐MPs exhibited good in vitro deposition efficiency and therapeutic efficiency on inducing LMBC apoptosis, which could be a novel inhalable integrated microsphere that provides a new possibility for diagnosis and thermoradiotherapy of LMBC.

## CONFLICT OF INTEREST

The authors declare no conflicts of interest.

## Data Availability

Research data are not shared.
